# Pregnancy, childbirth and neonatal outcomes of women with rare inherited coagulation disorders

**DOI:** 10.1177/1753495X221148813

**Published:** 2023-01-18

**Authors:** Lucy Jane McCann, Erin Scullion, Lauren Doy, Etienne Ciantar

**Affiliations:** 1 The Faculty of Medicine and Health, 4468 University of Leeds, Leeds, UK; 2 The Faculty of Epidemiology and Population Health, The London School of Hygiene and Tropical Medicine, London, UK; 3 Department of Obstetrics & Gynaecology, Leeds Teaching Hospitals NHS Trust, Leeds, UK

**Keywords:** Rare coagulation disorder, inherited coagulation disorder, post-partum haemorrhage, pregnancy

## Abstract

**Background:**

We aimed to describe the characteristics and the pregnancy outcomes of women with rare inherited coagulation factor disorders managed at a tertiary obstetric-haematology unit in the United Kingdom.

**Methods:**

A retrospective service evaluation was conducted using routinely collected medical records. Descriptive analyses were applied to investigate pregnancy, childbirth and neonatal management and outcomes.

**Results:**

Overall, 20 patients with rare inherited coagulation disorders were included who birthed 30 live infants from 29 pregnancies. Regarding maternal bleeding outcomes, 3% experienced antepartum haemorrhage, 38% of pregnancies experienced primary post-partum haemorrhage, and none experienced secondary post-partum haemorrhage. Five (17%) neonates had cranial ultrasound scans for imaging to investigate for a neonatal haemorrhage, which were all normal.

**Conclusions:**

Although women with rare inherited coagulation disorders may be more susceptible to complications in pregnancy, within this cohort there was no evidence that the condition led to increased morbidity or mortality when best practices were observed.

## Background

Pregnancy is a time of immense hemodynamic challenge, particularly for women with coagulation disorders.^1^ Rare coagulation factor disorders are defined as monogenic bleeding disorders which are a result of a deficiency in blood coagulation factors other than Haemophilia A, Haemophilia B and von Willebrand disease, and hence encompasses deficiencies of Factors II, V, VII, XI, XII and XIII as well as dysfibrinogenaemia.

Rare coagulation disorders represent 3–5% of all inherited bleeding disorders. According to the UK Haemophilia Centres Doctors’ Organisation, within the UK there are currently 7432 women registered with rare coagulation disorders.^2^

The haemostatic changes in pregnancy, and how they impact these women, remain uncertain. Whilst some factors appear to plateau or increase in pregnancy (FII, FV and FIX), others have been shown to decrease (Factors XI and XIII).^3^ Current evidence suggests that women with coagulation deficiencies may be at increased risk of maternal and neonatal mortality and morbidity throughout pregnancy, including bleeding during delivery, post-partum, and following terminations and miscarriages.^4,5^ Complications are not exclusive to the mother; fetal and neonatal complications can occur if the pregnancy is not managed optimally.^4^ However, the reliance on case studies has meant there is an unknown incidence of complications within this sub-group.

The Royal College of Obstetricians and Gynaecologists (RCOG) sets out evidence-based national guidance on the management of patients to optimise outcomes and standardise service delivery through the green-top guidelines. The current guideline states that the management of patients with coagulation deficiencies must be individualised and supported by a multidisciplinary team consisting of obstetricians, haematologists and anaesthetists.^1,6,7^ However, whilst extensive literature assesses the impact of Haemophilia A, Haemophilia B and Von Willebrand disease in pregnancy, there is a dearth of evidence focusing on rare coagulation deficiencies. This limits the guidance on the management of these patients. The lacking evidence is likely due to the rarity of the disorders and the ethical concerns with the conduction of higher-level evidence such as randomised controlled trials. Subsequently, pregnancy and childbirth can be uncertain time for patients and their managing practitioners.

The aim of this work is to describe the characteristics and outcomes of women with rare inherited coagulation factor disorders during pregnancy and birth managed at an obstetric-haematology unit in the UK from 1 September 2010 to 30 August 2021.

## Methods

Routine clinical maternal and neonatal records were retrospectively collected and analysed from all women with rare coagulation disorders whose births were managed in a tertiary university hospital trust between 2010 and 2021. No informed consent was required from patients due to the retrospective service evaluation design of the work.

Women who did not meet eligibility criteria were excluded from the analysis: births at a different hospital trust (*n*  =  10), no documented pregnancy in Leeds (*n*  =  8), or diagnosis of coagulation disorder after the birth (*n*  =  11). To maximize patient numbers due to the rarity of the coagulation deficiencies, the exclusion criteria were minimized where possible. For example, patients were not excluded due to comorbidities, maternal age or language barriers.

Initially, a proforma was generated to encapsulate all desirable variables in a standardized manner. To ensure accuracy in data collection, information on each eligible patient was cross-checked by two researchers. Then, descriptive analysis was conducted using Excel and used to report background maternal characteristics and maternal, birth and neonatal outcomes for each case. To follow standard statistical methodology, variables were presented as means (and standard deviations) and medians (and interquartile ranges (IQR)) for normally distributed and skewed variables, respectively.

The categorization of bleeding disorders into partial, mild and severe was defined by the trust policy of assessing bleeding phenotype by a haematology clinician. This took into consideration the patient's bleeding phenotype as well as the factor levels, bearing in mind that on occasions the two do not correlate. When this is the case, a greater emphasis is given to the bleeding history. Post-partum haemorrhage (PPH) was defined according to estimated blood loss as per the clinician's notes. Primary PPH was defined as an estimated blood loss of more than 500 mL within 24 h of birth as per the World Health Organization (WHO) definition.^8^ Secondary PPH was defined as excessive blood loss or heavy lochial discharge after 24 h of giving birth.

## Results

In total, 20 patients were eligible to be included who had 29 pregnancies managed at the hospital trust which resulted in 30 live births.

### Patient demographics

Table 1 presents the basic demographic characteristics and coagulation factor disorders of the patients included. The median maternal age at the time of birth was 30 years (IQR: 25–34). Thirteen patients were recorded as having previous pregnancies. There was only genetic genotyping information for three patients: two with severe Factor VII deficiency (both homozygous for c.1109G > T pathogenic variant in FVII gene) and one with severe Factor XI deficiency (homozygous for the P.(CYS416TYR), c.1247 G > A missense mutation in exon 11 of the FXI gene).

**Table 1.** The demographic characteristics and coagulation factor disorders of the patients.

Characteristics	Number, *n* = 20 (%)
**Age at time of first birth in Leeds**	
< 20	1 (5)
20–25	6 (30)
26–30	3 (15)
31–35	7 (35)
36–40	2 (10)
> 40	1 (5)
**Ethnicity**	
White: British	13 (65)
Asian: Pakistan	3 (15)
Asian: Chinese	1 (5)
Asian: Indian	1 (5)
Asian: Unspecified	1 (5)
Not documented	1 (5)
**Coagulation disorder**	
Dysfibrinogenaemia	4 (20)
Factor II deficiency	0 (0)
Factor V deficiency^a^	2 (10)
Factor VII deficiency	4 (20)
Factor X deficiency	0 (0)
Factor XI deficiency^a^	10 (50)
Factor XIII deficiency	0 (0)
**Coagulation severity**	
Mild	13 (65)
Partial	2 (10)
Severe	4 (20)
Not stated	1 (5)
**Bleeding history** ^b^	
No symptoms	5 (25)
Epistaxis	6 (30)
Bruising	6 (30)
Heaving bleeding post-miscarriage	2 (10)
Menorrhagia	10 (50)
Gum bleeding	2 (10)
Excessive bleeding post-surgery	1 (5)
Non-specified	2 (10)
**Miscarriage** ^b^	
0	11 (55)
1	4 (20)
2	2 (10)
3	2 (10)
Undocumented	1 (5)

^a^ One woman had a combination of VWD and Factor V, and one woman had VWD and Factor XI.

^b^ Some women experienced multiple symptoms.

The most common outcome of previous pregnancy was a miscarriage (14) followed by live births (11) and terminations (5). Of the 11 live births, four were forceps deliveries, four were spontaneous vaginal deliveries and three were via emergency caesarean section. Regarding miscarriages, over half of the women (11) had no previous known miscarriages (median: 0, IQR: 0-1). Two patients had recurrent miscarriages (defined as the loss of three or more consecutive pregnancies) but one also had a balanced translocation and uterine septum – conditions which predispose women to miscarriages. The other patient had two early miscarriages (< 6 weeks) and a missed miscarriage (at 12 weeks), with no reason for the miscarriages. However, she had no complications with her live birth.

### Birth and maternal outcomes

Table 2 presents the birth and post-partum management and outcomes of each pregnancy. Of the vaginal deliveries, 8 (40%) were induced due to a variety of reasons: reduced fetal movements (*n*  =  1), the prolonged second stage (*n*  =  1), recommended due to bleeding disorder (*n*  =  2) and undocumented (*n*  =  4). Both of the women whose labour was induced due to their factor deficiency had Factor XI deficiency. All women had serial haemoglobin testing and most had ferritin levels checked within the pregnancy, aligning with hospital guidelines to check full blood count and ferritin levels at 12 and 28 weeks of gestation. Those with low ferritin levels (five women (25%)) were given iron tablets, aligning with procedures to commence oral iron supplementation where there is evidence of iron deficiency anaemia or non-anaemic iron deficiency.

**Table 2.** The birth and post-partum outcomes of each pregnancy.

Outcome	*N* (%)	Additional comment
**Mode of birth (*n* = 29)**		
Vaginal delivery	20 (69)	
Caesarean	9 (31)	
**Intervention in NVD (*n* = 20)**		
Forceps	4 (20)	Three of the forceps deliveries were for one woman
Vacuum extraction	0 (0)	
Induced	8 (40)	
Complications	0 (0)	
**Analgesia (*n* = 24)**		
Nil or Entonox^®^	10 (42)	
Spinal	10 (42)	Those with Factor XI deficiency were covered with Octoplas^®^ but no other subgroups received any blood cover
General anaesthesia	3 (13)	
Epidural	1 (4)	No empirical therapy given prior to epidural analgesia and no documented complications from this
**Blood loss (*n* = 29)**		
Antepartum haemorrhage	1(3)	Women with factor XI deficiency and VWD type
0–499 mL	18 (62)	
500–999 mL	9 (31)	
1000 mL +	2 (7)	One was 1300 mL in a woman with mild dysfibrinogenaemia birthing twins, the other was 2200 mL in a woman with mild Factor XI deficiency
Secondary post-partum haemorrhage	0 (0)	
**Blood products given (*n* = 29)**		
Blood transfusion	1 (3)	Woman with Factor XI deficiency given two units of blood after 2200 mL PPH due to third-degree perineal tear when birthing twins
Octoplas^®^	8 (28)	50% of women with Factor XI deficiency were given Octoplas^®^
Fibrinogen	1 (3)	Women with dysfibrinogenaemia given 6–7 g of fibrinogen after 700 mL PPH

**Figure 1.**
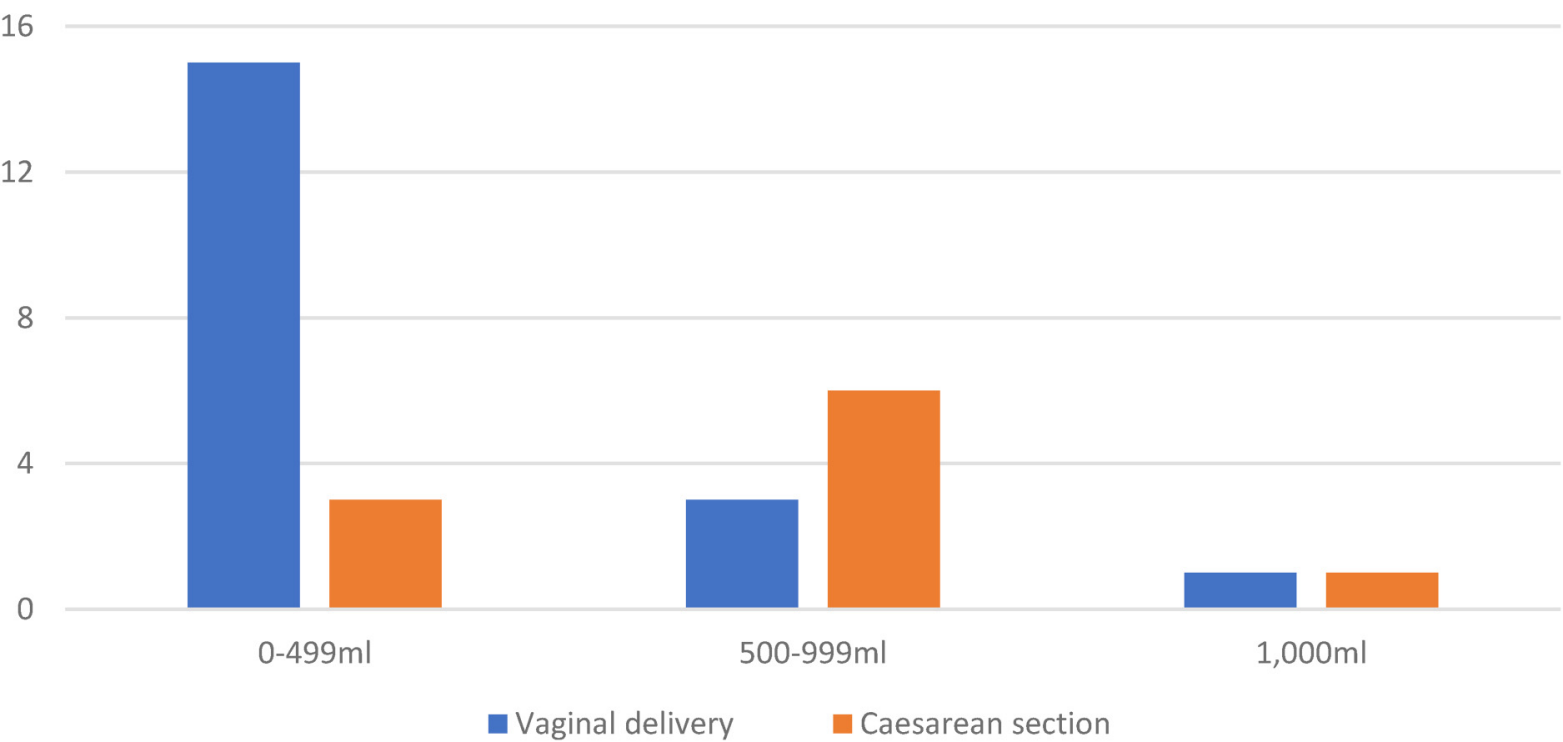
Primary post-partum haemorrhage (PPH) by delivery method.

Of the 11 (38%) pregnancies which experienced primary PPH, the majority were those delivered by caesarean section (*n*  =  7, 64%) (Figure 1). Baseline Factor XI was documented in seven of the pregnancies complicated by PPH and ranged from 27 to 58 units/dL (median: 35, IQR: 33–56). An antepartum haemorrhage of 400 mL occurred in 1 (3%) of the pregnancies who had Factor XI deficiency and VWD and were admitted with bleeding and abdominal pain at 40  +  0 weeks. Although an abruption was considered, the CTG was normal (HR  =  130, accelerations present, variability > 5 beats per minute). The patient was 2 cm dilated on vaginal examination and went into spontaneous labour. The baby was delivered via spontaneous vaginal delivery. After birth, there was a PPH of 600 mL followed by a further 200 mL bleed, totalling 1200 mL of blood loss. The bleeding was controlled with intramuscular oxytocin and there were no further complications to the mother or neonate.

The most significant major PPH was 2200 mL in a woman with Factor XI deficiency after a third-degree tear and atonic uterus. She received solvent/detergent treated, pooled human plasma (Octoplas™) and two units of blood, and subsequently, her haemoglobin postnatally was recorded as 124 g/L with no further bleeding complications.

In terms of blood products, the only women who received blood products were those with Factor XI deficiency and dysfibrinogenaemia (Table 2). Apart from the woman with a 700 mL PPH, no other women with dysfibrinogenaemia were given any blood products. Eight of the 16 pregnancies (50%) of Factor XI were managed using Octoplas^®^ due to post-partum bleeds or caesarean section delivery coverage. One woman refused Octoplas^®^ despite a 500 mL PPH as she self-discharged but this resulted in no further complications. One Factor XI pregnancy was managed with two units of blood after a third-degree perineal tear and a PPH of 2200 mL. No further complications were noted.

Tranexamic acid was documented as being prescribed in around half of the pregnancies (14, *n*  =  49%), which was always given as 1 g three times a day for 7–10 days post-partum or until lochia had stopped. In one pregnancy, tranexamic acid was used for 3 weeks postnatally until the per vaginum bleeding stopped. The use of paper notes for some means more may have been prescribed tranexamic acid but it was not documented.

### Neonatal outcomes

Within this cohort, all patients had pregnancies which carried to term (Table 3). All neonates weighed between 2500 and 4000 g apart from two which were 2380 and 2495 g. Five neonates (17%) had cranial ultrasound imaging scans to investigate for neonatal haemorrhages as per haematologist request These were all conducted in cases where the maternal coagulation deficiency was classified as ‘severe’ Factor XI or Factor VII deficiency. All of these scans were normal and no subsequent investigations or complications were documented. There was no evidence of any abnormal development in any of the neonates, except for one neonate being diagnosed with congenital hypothyroidism picked up on screening.

**Table 3.** Neonatal outcomes of each pregnancy.

Outcome	Median (IQR)	Additional comment
**Birthweight**	2455 g (2930–3605 g)	
**Gestation (weeks)**	39 (38–39)	
**Does the neonate have the deficiency? (*n* = 30)**	*N* (%)	
Yes	4 (13)	
Heterozygous	6 (20)	
No	7 (23)	
Unknown	13 (43)	
**Complications/investigations**		
Cranial ultrasound imaging	5 (17%)	All were reported as normal
Neonatal unit care post-birth	1 (3%)	For phototherapy for neonatal jaundice
Congenital hypothyroidism	1 (3%)	
Neonatal haemorrhage	0 (0%)	
**Vitamin K**		
Oral	15 (50%)	
Intramuscular	1 (3%)	
Unknown route	2 (7%)	
Undocumented	12 (40%)	

In terms of management, we found no evidence that a fetal scalp electrode was used during any of the pregnancies, as per the standard intrapartum recommendations. Almost two-thirds (60%) of the neonates were recorded to have been given vitamin K.

## Discussion

This audit collated evidence on the pregnancy management and outcomes of women and their offspring with rare inherited coagulation deficiencies managed within an obstetric-haematology department in a tertiary university hospital.

### Birth and maternal outcomes

Within this cohort of women, primary PPH occurred more commonly at 38% compared to the UK average obstetric cohort of 22%.^9^ These findings are consistent with a retrospective analysis by Clarke et al.^5^ in Australia which also found an increased frequency of PPH compared to the Australian national average. Although our cohort had a slightly higher frequency of bleeding, the estimated blood loss was smaller – the majority of the women experienced ‘minor’ bleeds of 500–1000 mL, with only two of the 11 primary PPH in our study having moderate or major bleeds of over 1000 mL compared to almost half in the study by Clarke et al. This disparity may be because Clarke et al. included women with common inherited bleeding disorders (VWD and haemophilia) in addition to those with rare bleeding disorders. Subsequently, our findings may be reassuring to those with rarer bleeding disorders in that, although the PPH risk may be increased, it does not seem to do so to a significant degree. In addition, all of the bleeds were adequately managed by routine measures such as intramuscular oxytocin, Octoplas™, factor replacement, or (in one case) blood transfusions.

The number of previous miscarriages seen within this small cohort does not suggest an increased risk of miscarriage. Over half of the cohort (55%) had not experienced a miscarriage, which is congruent with a recent study which found 43% of women reporting having at least one miscarriage within the general population.^10^ Whilst two women (10%) experienced three miscarriages, the clear documentation of balanced translocation and uterine septum within one of these pregnancies makes this the most likely cause. Whilst the cohort size must be considered when using this information in preconception counselling, these findings are still reassuring. This aligns with the current literature on rare inherited bleeding disorders and fetal loss, which currently shows that only FXIII deficiency is associated with fetal loss and placental morbidity.^11^

Notably, none of the women with ‘severe’ coagulation deficiencies experienced a PPH, suggesting that the risk of PPH may be difficult to predict based on coagulation severity alone and also difficult to separate from bleeding related to obstetric issues (e.g. uterine atony or perineal trauma). Notably, these values were only estimated blood loss, which is known to be less accurate than quantitative measurements of blood loss.^12^ In general, literature suggests that visual estimation of blood loss is more likely to underestimate blood loss, which may mean rates of PPH were higher within this group.^11^ However, within this rare cohort, they may have been overestimated as clinicians may be concerned about the patient having a coagulation deficiency and thus want to be more cautious. Nevertheless, it is important to take this finding with prudence. Aligning with the RCOG guidelines, all women with identified risk factors should have their delivery in a hospital with a blood bank on site.^13^

Only one pregnancy (3%) was complicated by an antepartum haemorrhage. As antepartum haemorrhage complicates 3–5% of pregnancies within all pregnancies in the UK, our findings suggest that bleeding disorders have had no impact on the likelihood of antepartum haemorrhage.^6^ This contrasts with previous literature which has suggested that women with rare coagulation deficiencies may be at increased risk of antepartum haemorrhage.^6,14^ However, these conclusions are based on case studies due to the rarity of the diseases and therefore subject to publication bias. The small sample size in our audit must be noted and unlikely has sufficient power to detect true differences. Despite this, our findings are still reassuring and contribute to the sparce evidence within this group.

Regarding the mode of delivery, no method was preferred, with 69% of babies delivered vaginally. This is similar to that seen in the UK population of around three in four.^15^ The requirement for a caesarean section should be dictated by obstetric reasons.

In terms of blood products, women with Factor XI and dysfibrinogenemia were the only ones given any during or after birth. Seven of the 16 (44%) pregnancies in Factor XI had a PPH, including the largest bleed of 2200 mL. Subsequently, this was the largest group to receive blood products; 50% of the pregnancies in women with Factor XI required Octoplas^®^, this is unsurprising as Factor XI is the only factor that does not increase physiologically in pregnancy.^6^ Therefore, of all the rare coagulation deficiencies, it is expected that those with Factor XI are most likely to receive fresh frozen plasma (FFP) to top up their levels. Tranexamic acid should not be given simultaneously with FFP.^6^ This additional requirement for FFP in these births means they should be prioritized at tertiary centres, where blood products are readily available.

### Neonatal outcomes

Regarding neonatal outcomes, almost all of the babies were born between 37 and 40  +  6 weeks of gestation (at term). This will optimise outcomes by minimising complications associated with pre and post-term births. A few births were induced to reduce the amount of post-terms, which produced no negative outcomes in these women. The birthweight of all babies was between 2500 and 4000 g apart from two who were low birthweight (as defined by the WHO as being less than 2500 g irrespective of gestational age). No babies were large for gestational age, defined as being over 4000 g.^16^

In terms of neonatal management, there was no evidence that a fetal scalp electrode was used during any of the deliveries. This shows management has aligned with the RCOG recommendations to avoid its use in neonates at risk of coagulation deficiencies. 18 (60%) of neonates were recorded to have been given vitamin K, the majority of which (83%) specified it was given orally. The limited documentation, for example, regarding whether vitamin K was given and the mode of delivery (oral or intramuscular), poses concerns. Oral vitamin K to neonates is recommended in neonates of unknown coagulation status as the standard route of intramuscular injection may introduce complications for these neonates.

### Maternal characteristics and generalizability

The maternal age of 31 was similar to national figures of the ages of mothers at childbirth in England and Wales.^17^ However, the ethnicity of patients varied slightly from Leeds demographics – this audit contained a lower proportion of White British (65% compared to 81.1% in the Leeds census 2021), and a higher proportion of Asians (20% compared to 7.7% in Leeds).^18^ This difference is likely because coagulation deficiencies are known to be more prevalent in Asian populations.^19,20^ The selection of patients within a single tertiary obstetric hospital in Leeds minimizes direct generalizability to other regions or countries, where patient demographics and management teams may vary. The demographic characteristics outlined in Table 1 will help compare with other populations.

### Strengths and limitations

The most notable limitation of this analysis is the limited number of eligible patients due to the rarity of the coagulation factor disorders and the inability to access records of patients outside of our NHS Trust. This has limited the scope for subgroup analysis and further statistical analysis of the data which reduces the reliability of the conclusions found. However, the large time period and substantial trust size we have used have maximized numbers, which still makes it one of the largest studies of rare coagulation deficiencies in the literature to date. The retrospective design has led to missingness. However, non-differential misclassification bias was minimized by using a standardized proforma for data collection which had fixed classification criteria. The use of quantitative and categorical answers reduced the discrepancy between data collectors. The accuracy of data collection was further enhanced by both researchers checking the proforma for every patient included.

### Future recommendations

From these findings, we have generated three recommendations to aid future care and research into women with rare coagulation deficiencies in pregnancy. Firstly, we recommend that antenatal care should be managed initially in a tertiary hospital within an obstetric haematology unit. Guidance from within this hospital should then dictate where the birth should be planned, considering the individual patient demographics and history. For example, patients with Factor XI or more severe bleeding disorders may need to have their delivery in tertiary units for ease of access to blood products and specialist baby units. In general, the outcomes for both mother and neonate were minimal and hence uncomplicated patients should be reassured they are safe to deliver at their most convenient hospital.

Secondly, we recommend that documentation of pregnancy and birth is improved. We suggest a proforma would be most appropriate to minimise missingness in the patient notes. For instance, ensuring a tick-box is added to the document with vitamin K has been given orally. This may serve as a prompt to remind clinicians that oral administration is preferable in neonates at risk of coagulation disorders. Tick-box forms have been proven to improve the quality of documentation, however, their impact on clinical behaviour is debated.^21^ Hence, regular audit and re-audit are necessary to ensure standards are optimised.

Finally, we suggest that linkage of electronic databases between trusts to help access of results. The limited sharing of data between trusts has led to the limited availability of data to be seen by consultants and researchers at obstetric haematology units. In addition to limiting the availability of data collection for this audit and other audits, it may also negatively impact patient care if clinicians are trying to provide advice to patients at other trusts.

## Conclusion

Although women with rare coagulation deficiencies may be more susceptible to PPH, within this cohort there was no evidence that their condition led to increased long-term morbidity or mortality when managed according to best practices. Women with rare coagulation deficiencies should be reassured that with appropriate management their outcomes are optimistic.
